# Determinants of malaria infection across different districts of Khyber Pakhtunkhwa, Pakistan: a cross-sectional study

**DOI:** 10.1186/s12936-025-05731-w

**Published:** 2026-01-24

**Authors:** Ijaz ul Haq, Zafar Mehmood, Amir Muhammad, Sohail Akhtar, Elmuez Alsir Ahmed Aboagarib, Afia Zaib, Sara Awadelkarim Mohammed Ahmed, Mashael Huwaikem, Gausal Azam Khan, Humood Fahm Albugami, Bilal Ahmed, Shenqiang Qu

**Affiliations:** 1 Department of Clinical Nutrition, College of Applied Medical Sciences, King Faisal University, Al Ahsa, Saudi Arabia; 2 Department of Math’s, Stats & Computer Science, The University of Agriculture Peshawar, Peshwar, Pakistan; 3 Pediatrics, Department, Lady Reading Hospital, Peshwar, Pakistan; 4 Department of Health Information Management and Technology, College of Applied Medical Sciences, King Faisal University, 31982 Al-Ahsa, Saudi Arabia; 5 Health Department, District Health Office, Battagram, Khyber Pakhtunkhwa Pakistan; 6 Department of Medicine, Maternity and Children’s Hospital, Al-Ahsa, Saudi Arabia; 7 Department of Public Health, College of Applied Medical Sciences, King Faisal University, Al Ahsa, Saudi Arabia; 8 School of Medicine, Qilu Institute of Technology, Jinan, Shandong, 2500000, PR China; 9 Department of Clinical Nutrition, Nanjing Tongren Hospital, Nanjing, China

**Keywords:** Malaria, Vector borne disease, Determinants, Transmission, Infectious diseases

## Abstract

**Background:**

Malaria remains a significant public health challenge, particularly in the Khyber Pakhtunkhwa (KP) province, where transmission patterns vary across districts. This study aimed to identify the determinants of malaria transmission using model selection techniques.

**Methods:**

A cross-sectional study was conducted among general population across various districts in KP, including northern, central, and southern zones. A total of 768 participants across the province were surveyed using a structured questionnaire having socio-demographic variables, housing conditions, access to healthcare, and preventive practices. Malaria infection was diagnosed using the combo rapid diagnostic test. Logistic regression models, including automated model selection techniques, were employed to identify significant predictors of malaria.

**Results:**

The overall proportion of malaria among respondents was 24.5%. Bivariate analysis showed that male gender, younger age (16–30 years), joint family system, low education, poor housing conditions, low income, long distance to health centers, and non-use of bed nets were significantly associated with malaria (p < 0.05). Final multivariate model results identified gender (AOR 1.8, *CI* 1.1–2.8, P < 0.05), age group 16–30 years (AOR 4.2, *CI* 2.1–8.5, P < 0.01), age group 31–50 years (AOR 8.3, *CI* 4.2–16.3, P < 0.01), age group > 50 years (AOR 3.6, *CI* 1.9–6.7, P < 0.01), family type (AOR 2.6, *CI* 1.47–4.61, P = 0.001), education level (AOR 3.9, *CI* 2.6–5.9, P < 0.01), income level (10,000–50000 rupees) (AOR 17.8, CI 8.1–38.9, P < 0.05), large family size (AOR 11.3, *CI* 5.9–21.7, P < 0.05), distance of > 3 km to healthcare facilities (AOR 3.1, *CI* 1.8–5.3, P < 0.01), and lack of modern toilets (AOR 4.8, *CI* 2.4–9.4, P < 0.01) as independent risk factors of malaria prevalence.

**Conclusion:**

The study highlights multiple risk factors contributing to malaria prevalence in KP. Tailored interventions, including improved access to education, healthcare, sanitation, and preventive tools like bed nets, are critical. Future studies incorporating geographic mapping and seasonal trends are recommended to strengthen targeted malaria control strategies.

**Supplementary Information:**

The online version contains supplementary material available at 10.1186/s12936-025-05731-w.

## Introduction

Infectious diseases remain significant challenges in the field of global health, ones that require careful thought and multifaceted approaches [1]. Since infectious diseases like malaria affect millions of people worldwide and have a considerable economic impact on the areas in which they occur, they pose a severe threat to public health [2]. Due to the global spread of its vectors, malaria is recognized as a mosquito-borne illness that spreads quickly and has a significant impact on morbidity and death,as, it poses a global public health concern [2, 3]. As per the WHO report 2022, there was an endemic prevalence of malaria in Pakistan between January and August 2022, with over 3.4 million suspected cases registered [4], as opposed to 2.6 million cases reported in 2021. Approximately 170,000 instances were confirmed in a lab setting, with 77% of those cases being caused by Plasmodium vivax and 23% by Plasmodium falciparum, which is linked to the deadliest and most severe cases [5]. According to the World Health Organisation, 84% of malaria cases in Pakistan are caused by P. vivax, with P. falciparum accounting for 14.9% of cases and mixed cases (double infections with P. vivax and P. falciparum) for 1.1% of cases [6].

Despite all of the disease control measures that the relevant authorities have developed and implemented, malaria still has a substantial impact on developing countries like Pakistan [7]. Malaria is more common disease in the tropics and subtropical regions [8]. Proper and early detection of malaria help in the reduction of transmission [9]. Human Plasmodium infections can present with a broad spectrum of symptoms, from mild malaria to severe cases. The 2015 WHO criteria for severe malaria include the following symptoms: hypoglycemia, jaundice, pulmonary edema, hemorrhage, numerous convulsions, prostration, acute renal failure, metabolic acidosis or acute respiratory distress, severe anemia, and pulmonary edema [10].

The occurrence, transmission, seasonality, and periodicity of malaria in particular—as well as the geographical and temporal distribution, severity, and duration of infectious diseases in general—are all influenced by socio-environmental factors [11].Understanding malaria dynamics in the context of local socioeconomic and meteorological conditions helps identify the significance of local variables driving malaria variability across time and space [12]. Poorly constructed homes allow easy entry for vectors carrying Plasmodium, increasing the risk of infection for household members. Low-income individuals often struggle to afford medications, related medical expenses, insecticide-treated bed nets (in countries where they are not distributed free of charge), and chemicals for indoor spraying [13].

There are numerous challenges in combating recurrent malaria in Pakistan. It is essential to assess the risk factors associated with malaria across different localities in Khyber Pakhtunkhwa (KP), Pakistan. Therefore, we conducted a study with the primary objective of identifying the determinants contributing to malaria transmission. The findings will help communities adopt effective preventive measures and will provide policymakers with valuable insights to develop informed strategies for malaria prevention in KP.

## Methods

### Study design and settings

The cross-sectional study was conducted from Jan 2022- Dec 2022 in selected districts of KP. This community-based study involved respondents from various districts of KP, including Dera Ismail Khan, Bannu, Karak, and Tank (south zone); Haripur, Abbottabad, Dir, Kohistan, Swat, and Chitral (north zone); and Peshawar, Charsadda, Nowshera, and Mardan (central zone). Inclusion criteria included participants who were willing to participate, available at home at the time of data collection, from any age group, and with no restrictions based on fever history.

### Sample size and sampling strategy

A representative sample of respondents was selected from various districts of KP. We used a two-stage sampling design. In the first stage, Primary Sampling Units (PSUs) were selected using probability proportional to size (PPS) sampling, purposively considering the severity of the disease and the prevailing law and order situation. In the second stage, Secondary Sampling Units (SSUs) were identified for the selection of index respondents. SSUs were selected using random-cum-systematic random sampling. The sample size was calculated using a formula.

($n={z}^{2}\left\{pq\right\}/{e}^{2}$), yielding an initial sample size of 384. However, considering the design effect (n x d), the final sample size was 768[14]. Design effect quantifies how much the actual sampling design increases or decreases the variance of your estimate compared to simple random sampling. A design effect of 2 was applied to the sample size obtained from the sample size formula to account for cluster sampling, which is a commonly used threshold value at a 95% confidence interval [14]. The sample was allocated Supplementary Table 1. The allocation of samples for socio-economic and demographic data was based on district population and reported malaria cases. Districts with higher populations and disease incidence were assigned larger samples, while those with lower malaria prevalence received smaller sample allocations.

### Data collection procedure

A pretested, structured questionnaire adapted from the WHO core standard questionnaire was used to collect data on socio-economic and demographic factors related to malaria [14]. Trained enumerators collected data by Kobo App (https://www.kobotoolbox.org/).

### Definition of malaria and study variables

Malaria was defined as a measured fever of ≥ 37.5 °C within the past 48 h accompanied by a positive blood smear for Plasmodium falciparum [15]. To confirm a malaria case, combo RDTs and microscopy were used interchangeably, depending on their availability at the laboratory. Explanatory variables included socio-economic and demographic characteristics such as age, sex, marital status, family size, education, occupation, family income, household type, distance to the nearest health center, water source, bed net availability and use, latrine availability, kind of utensils used for storing water, drinking water quality, and availability of any kind of livestock in home [14].

### Statistical analysis

Data were checked, verified, and analyzed using statistical software (R). Descriptive statistics, odds ratios, and logistic regression models (binary and multiple) were used to analyze the data.

A household-level random effect was included because more than one participant could be sampled from the same household. To account for variability across districts, a district-level random effect was also incorporated into the multivariable logistic regression model. The logistic regression model was used to examine the relationships between the binary dependent variable and socio-economic variables. Automated model selection method using R with ${\alpha }_{in}=0.07 and {\alpha }_{out}=0.18$ were used for the final determinants of malaria**.** A p-value < 0.05 was considered significant.

## Results

### Demographic and socio-economic characteristics of the study population

Table 1 displays the percentage distribution of socio-economic variables. The total number of individuals included in the study were 768. The majority (69.4%) were males, 34.5% were between 30 to 50 years of age, 39.1% lived in semi-pucca houses (partially, 54.6% lived in nuclear families, and 46.2% had a small family size. Regarding education, 44% were below secondary school certificate (SSC). As for other characteristics, 55% were self-employed, the majority (37%) of respondents lived at a distance of 1–3 km from the nearest health center, 47.1% of households had an income above 50,000 rupees, 44.3% kept livestock, 66.9% had a safe flush toilet, and only 36.3% of respondents used bed nets while sleeping outdoors.

**Table 1:** Percentage distribution of socioeconomic and demographic variables of the study population

Characteristics		N	%
Gender	Male	533	69.4
	Female	235	30.6
Age (in years)	< = 15	147	19.1
	16–30	166	21.6
	31–50	265	34.5
	> 50	190	24.7
Family Size (individuals)	Small (1–5)	355	46.2
	Medium (5–8)	224	29.2
	Large (> 8)	189	24.6
Family Type	Joint	349	45.4
	Nuclear	419	54.6
Marital Status	Unmarried	375	48.8
	Married	393	51.2
Education of the Respondents	< SSC	338	44.0
	= > SSC	430	56.0
Occupation	Peasant	173	22.5
	Employed	138	17.9
	Self-employed	423	55.0
	Un-Employed	35	4.5
Income (RS)	< 20,000	233	30.3
	20,000–50000	173	22.5
	> 50,000	362	47.1
Availability of any Kind Of Livestock at household	Yes	340	44.3
	No	428	55.7
House Type	pucca	178	23.2
	Semi pucca	300	39.1
	Kacha	290	37.8
Modern Toilet facility available	Yes	514	66.9
	No	254	33.1
Kind Of Utensils Use For Storing Water	Without Lid	289	37.7
	With Lid	479	62.3
Drinking Water Quality	Not Improved	365	47.5
	Improved	403	52.5
Bed Net Use	No/Partial	489	63.7
	Yes	279	36.3

Supplementary table shows the proportion of participants malaria positive in each district. The results revealed that proportion of malaria was 24.5% (n = 188) out of total 768 respondents.

### Bi-variate logistic regression analysis for malaria and its associated risk actors

Bivariate logistic regression analysis with odds ratios is given in Table 2. The results revealed that males were 1.73 times more likely to suffer from malaria compared to females (OR = 1.73 (1.18–2.55), P < 0.001), and individuals aged 16 to 30 years were 5.20 times more likely to suffer from malaria compared to the age group greater than 50 years (OR = 5.20, P < 0.001). The odds of malaria were about 1.43 times higher in individuals living in a joint family system (OR = 1.43 (1.03–1.99), P < 0.05), while the odds of malaria were about 4.59 times higher in large family sizes compared to small family sizes (OR = 4.59 (3.02–6.99), P < 0.001). Similarly, those with no or below matric education were about 3 times more likely to suffer from malaria compared to individuals with matric or above education (OR = 3.12 (2.21–4.39), P < 0.001). Individuals living in houses constructed with semi/partial concrete materials were about 1.76 times (OR = 1.76 (1.15–2.69), P < 0.01) more likely to suffer from malaria, and those living in houses constructed with mud/kacha materials were about 1.2 times (OR = 1.21 (0.82–1.76), P > 0.05) more likely to suffer from malaria compared to individuals living in houses with full concrete/pucca construction materials. People with low income (i.e., income < Rs. 10,000) were about 3 times (OR = 3.02 (2.01–4.53), P < 0.001) more likely, and those with middle income (i.e., income Rs. 10,000–50,000) were about 3.5 times (OR = 3.49 (2.27–5.36), P < 0.001) more likely to suffer from malaria compared to individuals with income above Rs. 50,000. People living at a distance of 1–3 km and more than 3 km from the Nearest Health Center (NHC) were about 1.7 and 2.2 times, respectively, and significantly more likely to suffer from malaria compared to those living at a distance of below 1 km from NHC. Those who were not using bed nets were about 2.59 times (OR = 2.59 (1.76–3.81), P < 0.001) more likely to suffer from malaria compared to those using bed nets while sleeping.

**Table 2:** Results of binary logistic regression for Malaria

Characteristics		Malaria		OR(CI)	P-Value
		No	Yes		
Gender	Male	387	146	1.73 (1.18–2.55)	0.005
	Female	193	42	–	
Age (Years)	0–15	107	40	3.18 (1.76–5.73)	< 0.01
	16–30	103	63	5.20 (2.97–9.10)*	
	31–50	200	65	2.76 (1.61–4.75)*	
	> 50	170	20	–	
House Type	pucca	122	56	–	0.009 0.335
	Semi pucca	228	72	1.76(1.15–2.69)*	
	Kacha	230	60	1.21(0.82–1.76)^NS^	
Family Type	Joint	251	98	1.43(1.03–1.99)*	0.035
	Individual	329	90	–	
Family Size	Small	307	48	–	< 0.01
	Medium	163	61	2.39(1.57–3.65)**	
	Large	110	79	4.59(3.02–6.99)**	
Education	< SSC	216	122	3.12(2.21–4.39)	< 0.01
	> = SSC	364	66	–	
Occupation	Peasant	80	27	0.97(0.57–1.65)	> 0.05
	Employed	135	49	1.04(0.67–1.63)	
	Self employed	201	55	0.79(0.52–1.20)	
	Un-employed	164	57	–	
Income (Rs)	< 10,000	157	76	3.02(2.01–4.53)**	< 0.01
	10,000–50000	111	62	3.49(2.27–5.36)	
	> 50,000	312	50	–	
Availability of any kind of Livestock	Yes	246	94	1.36(0.98–1.89)	0.05
	No	334	94	–	
Marital Status	Married	298	95	1.03(0.74–1.44)*	0.83
	Unmarried	282	93	–	
Drinking Water Quality	Improved	310	93	–	0.34
	Not Improved	270	95	1.17 (0.84–1.63)	
Distance to the Nearest Health Center	< 1 km	184	37	–	0.026 0.001
	1–3 km	213	71	1.66 (1.06–2.58)	
	> 3 km	183	80	2.17(1.40–3.38)**	
Kind of Utensils Used for Storing Water	Without lid	210	79	1.96 (1.374–2.78)**	< 0.001
	With lid	370	109	–	
Bed Net Use?	Yes	239	40	–	< 0.01
	No	341	148	2.59 (1.76–3.81)	
Modern Toilet facility available	Yes	388	126	–	> 0.05
	No	192	62	0.99 (0.70–1.41)	

^*^Shows results significant at 5% level of significance

^**^Shows results significant at 1% level of significance

*NS* stands for Non-significant

### Stratified analysis of bed net uses by level of education on malaria status

The results of the stratified analysis of bed net utilization by education on the effect of malaria are presented in Supplementary Table 3. It is evident that individuals with no or less education are at a higher risk of being infected with malaria compared to individuals with SSC or higher education (OR = 1.67 for no or less than SSC education vs. OR = 1.10 for educated individuals). The analysis also showed a higher prevalence of malaria (39.2%) among suspected patients with no or less education who did not use bed nets, compared to educated suspected patients who also did not use bed nets during sleep (only 14.6%).

### Summary statistics for the final model of collected data

Automated model selection method with ${\alpha }_{in}=0.07\text{and }{\alpha }_{out}=0.18$ were used for the final determinants. Using the optimal cutoff values for entry and removal of predictor variables in the model, the final multiple logistic regression model is summarized in Table 3. The results of multivariate model identified gender (AOR 1.8, *CI* 1.1–2.8, P < 0.05), age group 16–30 years (AOR 4.2, *CI* 2.1–8.5, P < 0.01), age group 31–50 years (AOR 8.3, *CI* 4.2–16.3, P < 0.01), age group > 50 years (AOR 3.6, *CI* 1.9–6.7, P < 0.01), family type (AOR 2.6, *CI* 1.47–4.61, P = 0.001), education level (AOR 3.9, *CI* 2.6–5.9, P < 0.01), income level 10,000–50000 rupees (AOR 17.8, CI 8.1–38.9, P < 0.05), large family size (AOR 11.3, *CI* 5.9–21.7, P < 0.05), distance to healthcare facilities more than 3 km (AOR 3.1, *CI* 1.8–5.3, P < 0.01), and lack of modern toilets (AOR 4.8, *CI* 2.4–9.4, P < 0.01) as independent risk factors of malaria prevalence.

**Table 3:** Final model for the determinants of Malaria transmission

		B	S.E	Wald	df	Exp(B)	*95% CI*	*P*
Gender		0.586	.235	6.205	1	1.797	1.13–2.85	0.013
Age (years)	*0–15*							
	*16–30*	1.439	0.356	16.301	1	4.218	2.10–8.47	< 0.01
	*31–50*	2.115	0.345	37.642	1	8.290	4.21–16.32	< 0.01
	> *50*	1.268	0.327	15.041	1	3.555	1.87–6.74	< 0.01
Family Type		0.957	0.292	10.733	1	2.604	1.47–4.61	0.001
Education		1.362	0.214	40.366	1	3.905	2.57–5.94	< 0.01
Income (rupees)	< 10,000							
	10,000–50000	2.881	0.399	52.034	1	17.824	8.16–38.94	< 0.01
	> 50,000	1.783	0.276	41.606	1	5.946	3.46–10.22	< 0.01
Family Size	Small							
	Medium	1.820	0.346	27.611	1	6.173	3.13–12.15	< 0.01
	Large	2.429	0.330	54.085	1	11.344	5.95–21.70	< 0.01
Distance to the Nearest Health Center	< 1 km							
	1–3 km	0.655	0.275	5.671	1	1.925	1.12–3.30	0.017
	> 3 km	1.132	0.276	16.815	1	3.103	1.81–5.33	< 0.01
Modern Toilet facility available		1.560	0.347	20.272	1	4.760	2.41–9.40	< 0.01
Constant		− 9.539	0.906	110.813	1	0.000		< 0.01

## Discussion

The study demonstrated a significant association between malaria and various socio-demographic factors, including age and gender of the head of household, family size, family type, educational status, toilet availability, distance to nearest healthcare facilities, and income level. Furthermore, the proportion was 24.5% of malaria Khyber Pakhtunkhwa, highlighting the substantial burden on the health system.

The two common parasites in the study region, Plasmodium falciparum and Plasmodium vivax, impact individuals of all ages and genders [16]. In the current study, gender was a significant risk factor that influenced the frequency of malaria in a community. Women are comparatively more involved in interior domestic chores, while male heads of families are more likely to participate in outside activities, increasing their exposure to mosquitoes [17]. Many studies have explored the similar risk of getting malaria for both genders [18]. Our findings on the significant association between age and malaria status are consistent with Kigozi et al. (2020) [19]. While prior studies suggest that children under five are more likely to contract malaria [20]. other research indicates that early parental reporting, social lifestyle, knowledge, prevention, and maternal antibodies may contribute to lower malaria frequencies in children aged 1–3 [21].

The size of the family was one of the other extremely important socio-demographic factors that the current study examined. Compared to smaller families, households with more members or joint family had significantly greater odds of experiencing a malaria case, and these findings are consistent with the study reported by Thomas et. al [18]. This may be the case due to the higher likelihood of younger children in big households, who belong to a high-risk demographic. Because the olfactory signals for mosquitoes get stronger at crowding and attracting more insects, the number of occupants in a residence also increases the population of mosquitoes [22].

Another risk factor for the development of malaria was the distance between the home and the closest medical institution. According to the study's findings, family members who live close to health facilities tend to have more frequent and timely access to medical treatment than those who live far from such facilities. However, these findings are inconsistent with those of Toh et al. [23]. The distance to a health facility can be a barrier to regular health evaluations due to transportation costs and lost work time. The accessibility of a health institution is influenced by factors such as distance and transportation expenses.

The current research found a statistically significant relationship between participants' education level and malaria incidence. Respondents with lower levels of education and illiteracy were at a higher risk of contracting malaria, which is inconsistent with the findings of Siegert et al. [24]. This result was expected, as education improves knowledge of infectious diseases and necessary preventive measures. Education is thought to function as a protective social factor through various indirect pathways, including occupation, socioeconomic position, and symptom knowledge, which increases the likelihood of treatment-seeking behavior. However, a previous study in India found no association between education and malaria incidence [25].

Many studies have demonstrated a strong correlation between low income and malaria, which is commonly believed to be a disease of the poor. These findings are consistent with the current study [26]. However, other research has not shown a strong correlation between household income and malaria [17]. Additionally, malaria cases often increase after natural catastrophes, particularly in low-income nations like Pakistan [27]. The primary causes of this increase include inadequate vector control, inefficient surveillance systems, and poverty.

The study also investigated the relationship between malaria and toilets facility in households. Earlier research found that inadequate sanitation facilities represent a significant risk factor [28]. Another study found lack of toilet facility as a risk factor of malaria [29]. Another study reported that households with flush toilet had a decreased risk of malaria prevalence whereas no toilet facility increases risk of malaria [30].

The implementation of malaria control methods, such as bed nets greatly decreased the likelihood of contracting malaria. Use of bed nets prevents mosquito biting and thus prevents malaria. These findings are consistent with an earlier study [31]. However, some studies have found no correlation between the use of insecticide-treated nets, and long-lasting insecticide-treated nets [32]. In our study, bivariate analysis revealed a significant relationship between bed net use and malaria transmission, but this relationship was not significant in the final model.

There were certain limitations of the study. First, because of its cross-sectional study design, It may not show cause and effects. Second, as malaria in recurring in the region, mapping of the incidence is lacking. Therefore, in the future, we have planned to conduct study about the mapping of malaria for the targeted interventions. The altitude and latitude is also missing, which may show better interventions for the high and low altitude areas. Nevertheless, the current study has comprehensively identified the different risk factors of malaria by applying different statistical models.

## Conclusion

This study highlights the multifactorial nature of malaria transmission in KP, Pakistan, with strong associations found between malaria infection and various determinants. Key risk factors identified include younger age, male gender, low educational attainment, low household income, large family size, distance from health facilities, poor housing construction, and lack of modern sanitation. Although preventive measures such as bed net use showed significance in bivariate analysis, their independent effect was not retained in the final model, suggesting the need for comprehensive interventions beyond individual behavior change. Policymakers and public health authorities should use these findings to inform district-level malaria control strategies, focusing on education, improved housing, access to healthcare, and sanitation. Government along with International NGOs should work in collaboration for preventive strategies to control malaria in the region. Future research should incorporate geographic mapping, altitude data, and seasonal patterns to enhance the targeting and effectiveness of malaria prevention programs in the region.

## Supplementary Information

Supplementary file 1

## Data Availability

No datasets were generated or analysed during the current study.
