# Supplementary material for: Determinants of malaria infection across different districts of Khyber Pakhtunkhwa, Pakistan: a cross-sectional study
Source: Malar J. 2026 Jan 24;25:102. doi: 10.1186/s12936-025-05731-w (PMC12911161; doi:10.1186/s12936-025-05731-w)
Supplement: Supplementary file 1 [file 12936_2025_5731_MOESM1_ESM.docx]

**Supplementary Table 1. Sample distribution across the districts**

| **Districts** | | **Sample** |
| --- | --- | --- |
| 1 | Karak | 64 |
| 2 | Bannu | 85 |
| 3 | Dera Ismail Khan | 92 |
| 4 | Peshawar | 96 |
| 5 | Mardan | 54 |
| 6 | Nowshera | 56 |
| 7 | Haripur | 82 |
| 8 | Abbottabad | 68 |
| 9 | Swat | 66 |
| 10 | Dir | 58 |
| 11 | Chitral | 47 |
| **Total** | | **768** |

**Supplementary Table 2. Proportion of Malaria transmission Various Districts of KP**

| **District** | **Malaria Infection (Yes)**  **n (%)**  **N=188 (24.5 %)** | **Malaria Infection (No)**  **n (%)**  **580 (75.5 %)** |
| --- | --- | --- |
| **Karak** | 16 (2.0) | 48 (6.3) |
| **Bannu** | 21 (2.7) | 64 (8.4) |
| **Dera Ismail Khan** | 23 (2.9) | 69 (9.0) |
| **Peshawar** | 23 (3.1) | 73 (9.4) |
| **Mardan** | 13 (1.7) | 41 (5.3) |
| **Nowshera** | 14 (1.8) | 42 (5.5) |
| **Haripur** | 20 (2.6) | 62 (8.1) |
| **Abbottabad** | 17 (2.2) | 51 (6.7) |
| **Swat** | 16 (2.1) | 50 (6.5) |
| **Dir** | 14 (1.9) | 44 (5.7) |
| **Chitral** | 11 (1.5) | 36 (4.6) |

**Supplementary Table 3 : Stratified Analysis of Bet Net Uses by Level of Education on the effect of Malaria**

| ***Education*** | ***Bed Net Utilization*** | | ***Had Malaria*** | | | | | | | | ***OR(95% CI)*** |
| --- | --- | --- | --- | --- | --- | --- | --- | --- | --- | --- | --- |
|  |  |  | ***No*** | | | | ***Yes*** | | | |  |
|  |  |  | ***N*** | | ***N %*** | | ***N*** | | ***N%*** | |  |
| ***No or <SSC*** | ***Yes*** | **27** | | 100% | | **0** | | 0% | | 1.67 (1.51-1.80) | |
|  | ***No*** | 189 | | 60.8% | | 122 | | 39.2% | |  |  |
| ***SSC or Above*** | ***Yes*** | 212 | | 84.1% | | 40 | | 15.9% | | 1.10 (0.65-1.89) | |
|  | ***No*** | 152 | | 85.4% | | 26 | | 14.6% | |  |  |
